# Hydrothermal treatment: An efficient food waste disposal technology

**DOI:** 10.3389/fnut.2022.986705

**Published:** 2022-09-12

**Authors:** Xinyan Zhang, Qingyu Qin, Xun Sun, Wenlong Wang

**Affiliations:** ^1^National Engineering Laboratory for Reducing Emissions from Coal Combustion, Shandong Key Laboratory of Energy Carbon Reduction and Resource Utilization, School of Energy and Power Engineering, Engineering Research Center of Environmental Thermal Technology of Ministry of Education, Shandong University, Jinan, China; ^2^Laboratory of Biomass and Bioprocessing Engineering, College of Engineering, China Agricultural University, Beijing, China; ^3^Key Laboratory of High Efficiency and Clean Mechanical Manufacture, Ministry of Education, School of Mechanical Engineering, Shandong University, Jinan, China

**Keywords:** food waste, hydrothermal treatment, nutritional composition, physicochemical property, reuse

## Abstract

The quantities of food waste (FW) are increasing yearly. Proper disposal of FW is essential for reusing value-added products, environmental protection, and human health. Based on the typical characteristics of high moisture content and high organic content of FW, hydrothermal treatment (HTT), as a novel thermochemical treatment technology, plays unique effects in the disposal and utilization of FW. The HTT of FW has attracted more and more attention in recent years, however, there are few conclusive reviews about the progress of the HTT of FW. HTT is an excellent approach to converting energy-rich materials into energy-dense fuels and valuable chemicals. This process can handle biomass with relatively high moisture content and allows efficient heat integration. This mini-review presents the current knowledge of recent advances in HTT of FW. The effects of HTT temperature and duration on organic nutritional compositions (including carbohydrates, starch, lipids, protein, cellulose, hemicellulose, lignin, etc.) and physicochemical properties (including pH, elemental composition, functional groups, fuel properties, etc.) and structural properties of FW are evaluated. The compositions of FW can degrade during HTT so that the physical and chemical properties of FW can be changed. The application and economic analyses of HTT in FW are summarized. Finally, the analyses of challenges and future perspectives on HTT of FW have shown that industrial reactors should be built effectively, and techno-economic analysis, overall energy balance, and life cycle assessment of the HTT process are necessary. The mini-review offers new approaches and perspectives for the efficient reuse of food waste.

## Introduction

With the development of society and the improvement of living standards, people have higher requirements for the quality and quantity of food, which leads to the generation of a large amount of food waste (FW) (1). It has been estimated that 1.6 Gt of FW has produced annually around the world (2). Studies have shown that FW accounts for 50%−70% of municipal solid waste and more than 90% of the is currently discarded in landfills or incinerated in China (3). In Europe, around 20% of FW is treated by aerobic digestion and about 80% is composted (4). In the USA, nearly 58% of FW is treated by anaerobic digestion, and the rest is usually buried in landfills (5). A study has shown that weekly avoidable food waste per household resulted in economic losses of $ 18.1, nutritional losses of 3,366 calories, and 23.3 kg of CO_2_ emissions in Guelph and Ontario (6). Chalak et al. (7) have shown that household food waste constitutes a sizable proportion of the total waste generated throughout the food supply chain, and well-defined regulations, policies, and strategies are more effective than fiscal measures in mitigating household food waste generation. The traditional treatment methods of FW are disposed of in landfills (8) and composting (9), anaerobic digestion (10), and incineration (11), which will cause waste of resources and environmental pollution. Organics are the main kinds of substances in FW. Thermal conversion technologies, such as gasification, combustion, carbonization, and pyrolysis are commonly applied to organics treatment for waste-to-energy conversion. FW usually contains a lot of water, and these thermal conversion technologies usually require pre-drying of raw materials, which could increase energy consumption. Hydrothermal treatment (HTT) is no limit on the water content of feedstock (12, 13), so it show great advantages in the treatment of FW (14). In addition, the process of HTT can process energy-dense fuels and valuable chemicals and allow efficient heat integration (15). The HTT of FW can also achieve bactericidal action. According to the statistical analysis of the Web of Science, from 2012 to 2021, the number of articles on HTT of FW is 34, 30, 44, 59, 67, 104, 129, 162, 200, and 223, respectively. It indicates that the HTC of FW has gradually attracted the attention of researchers. The HTT-based FW has been investigated in recent years, however, there are few conclusive reviews on the progress of HTT of FW. Hence, the present mini-review summarizes the research progress of the current studies about HTT of FW, illustrates the characteristics and principles of HTT, and describes the effects of HTT on the nutritional compositions and the physicochemical properties of FW. The applications and economic analyses of HTT in FW are analyzed. The mini-review provides new approaches and perspectives for the efficient reuse of nutrients from food waste. This paper also provides economic and technical guidance for the utilization of food waste.

## Characteristics and principles of hydrothermal treatment

HTT refers to feedstock reacting in liquid media under high temperatures and corresponding pressure conditions, and the temperature range is usually between 100 and 700°C (16). With the increase in reaction degree, the HTT can be divided into hydrothermal carbonization (HTC), hydrothermal liquefaction (HTL), and supercritical water gasification (SCWG) (17, 18), and the main products will be transformed from the solid phase to the gas phase. Various hydrothermal processes depending on operating conditions have significant impacts on FW (Table 1). HTC temperature is usually 150–250°C, which generates the solid phase used for solid fuel or adsorption. HTL temperature range is 250–370°C and it is at pressures above the equilibrium vapor pressure of water, which can convert wet biomass into an energy-dense bio-oil using subcritical water. SCWG can occur when the temperature and pressure exceed the critical point of water (T > 374°C, P > 22.1 MPa) (31). And the SCWG process can produce combustible gases, namely, methane and hydrogen (32). The advantages, disadvantages, mechanisms, and processing treatments of HTT of FW are listed in the Supplementary Table S1 of the additional files. Studies have shown that HTT can be used to produce xylanases and lactic acid (33).

**Table 1:** Effects of hydrothermal treatment on food waste.

**Treatment method**	**Condition range**	**Physicochemical properties**	**Additional information**	**References**
HTC	T: 150–250°C	The pH increases with the rising reaction severity	Hydrochar yield of food waste with high proteins and fat is lower than that of food waste with high carbohydrates	(15, 19–24)
		The formation of soluble organic alkalis; the degradation of intermediated organic acid		
		The C content increases and N content decreases		
		With the increasing of HTC temperature, the hydrochar surface forms more acidic functional groups		
		Morphology and structure significantly change		
HTL	T: 250–370°C	Liquid phase products have nutrients and toxic substances	Produce biocrude oil	(25, 26)
		Solid phase products are rich in heavy metals and phosphorus		
SCWG	T: 600–700°C, P > 22.1 MPa	The water has high solubility for organic substances, reactivity and diffusivity, and low viscosity and permittivity	Generating syngas with a high H_2_	(27–30)
		The addition of catalysts (including NaCl, NaHCO_3_, NaCO_3_) can promote the gasification of food wastes, increasing H_2_ yield (420–480°C, 28 MPa, 5–15 WT%, 30–75 min)	The order of effect on H_2_ yield is temperature > feed concentration > residence time	
		Na^+^ can promote the SCWG of FW (400–450°C, ~25 MPa, 5 WT%, 20–60 min)	NaOH is the best catalyst among NaOH, NaHCO_3_, NaCl	
		The H_2_ yield is maximum at 500°C for 60 min, with the addition of 5 wt% KOH, the H_2_ yield increased by 52.7% (420–500°C, ~28 MPa, 2–10 wt%, 20–60 min)	KOH is the best catalyst among FeCl_3_, K_2_CO_3_, activated carbon, KOH	
			Alkali catalysts can promote the H_2_ production	

Compared with HTL and SCWG, HTC is a low-temperature HTT (1). HTC is the most widely used in the treatment of FW, followed by HTL and SCWG. HTC is an exothermal reaction and its products are mainly hydrochar with high energy content, good grindability, and high hydrophobicity. With the temperature increasing, due to the presence of many more ions, subcritical water has more reactive than water under ambient conditions to promote bond cleavage of the polymeric chain in organic waste (34). The HTC reaction mechanisms are usually six main procedures, including hydrolysis, dehydration, decarboxylation, condensation, polymerization, and aromatization (24, 35–37). During the process of HTC, catalysts can be applied to facilitate specific reaction pathways to enhance the characteristics of hydrochar with less energy consumption. In the acidic conditions of HTC, hydrogen ions can be released to improve the depolymerization processes of FW (38). Organic acids can provide an acidic environment to be applied for catalysts, and they are the main intermediates decomposed from monomers and polysaccharides (39).

HTL is a thermochemical process and can convert wet organic biowastes to renewable crude oil through high temperature and high pressure, which is an operating temperature of 300–350°C at 5–20 MPa (40). And crude oil can be used as a precursor to an efficient fuel. Moreover, during the process of HTL, the solid phase products are rich in heavy metals and phosphorus, while the liquid phase products have nutrients and toxic substances (25, 26). The process of HTL begins with the solvolysis of biomass in micellar forms, the disintegration of biomass fractions (cellulose, hemicellulose, and lignin), and thermal depolymerization into smaller fragments (33). At present, various operating conditions and feed-stocks have been applied for the production of crude oil through HTL treatment in some batch reactions and small continuous systems (41, 42).

SCWG is a thermochemical conversion method, that belongs to hydrothermal gasification. Its water is at a supercritical state in the range of 600–700°C, which has a high solubility for organic substances, reactivity and diffusivity, and low viscosity and permittivity (38). Supercritical water can efficiently decompose organic substances to generate syngas with a high H_2_, which is attributed to the elimination of interphase mass transfer limitations (27). In addition, the products from SCWG also include CH_4_, CO_2_, CO, and small amounts of C_2_H_6_ and C_2_H_4_. The efficiency of the SCWG process can be improved significantly by suitable catalysts to enhance the water-gas shift reaction (43). For example, H_2_ production can be promoted using alkali catalysts in the process of SCWG. The reaction temperature of HTT can be lowered by the addition of catalysts (38). The heterogeneous (Rh, Ni, Ru, etc.) and homogeneous catalysts (NaOH, Na_2_CO_3_, KOH, K_2_CO_3_, KHCO_3_, etc.) can be efficient in promoting the gasification reaction (33, 44). However, the homogeneous catalysts may cause salt deposition problems, and the heterogeneous catalysts has poor hydrothermal stability. Therefore, the rational use of catalysts can promote the hydrothermal reaction more efficiently. In addition, Karayildirim et al. (45) proposed that char and coke might be converted through solid-solid conversion and degradation-polymerization during the process of SCWG.

## Effects of hydrothermal treatment on the properties of food waste

The influence factors of HTT on FW include composition, HTT temperature, HTT residence time, and so on, among which composition and temperature are the main influential factors (22, 23, 46).

### Nutritional compositions

**Figure 1:**
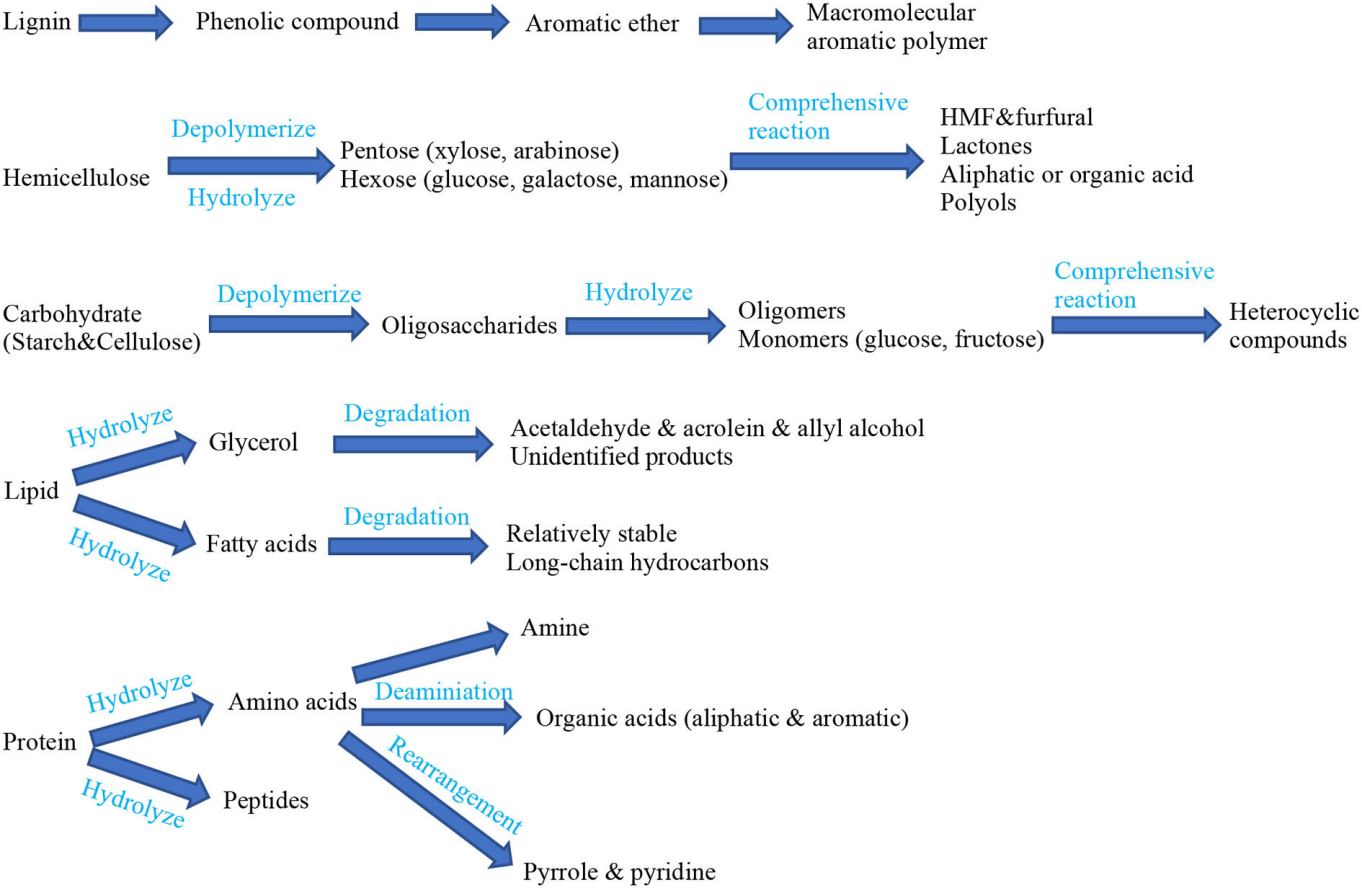
Pathways for decomposition of main components of FW in HTC process (1, 15).

The compositions of FW are complex, including carbohydrates, proteins, lipids and lignin, and so on. The compositions of FW can degrade during HTT so that the physical and chemical properties of FW can be changed. The decomposition pathways of the main organic nutritional components of FW in the HTC process are illustrated in Figure 1 (1, 15). Carbohydrates can be easily hydrolyzed during the process of HTC. They usually include starch, cellulose, and hemicellulose so the main intermediates are glucose and xylose. And the final products usually include HMF, lactones, polyols, and carboxylic acid. Lignin is difficult to hydrolyze. Lipids are difficult to dissolve in water, however, in the process of HTT, lipids can be hydrolyzed into glycerol and fatty acids under the action of supercritical water. Protein can be degraded into amino acids under HTT conditions and then into hydrocarbons, amines, aldehydes, and acids after further deamination and decarboxylation. These degradation products can be collected as basic synthetic materials to realize the resource utilization of nutrients in FW.

Due to the compositions of various FW being different, the final products show different properties after HTT. The hydrochar yield of FW with high proteins and fat is lower than that of FW with high carbohydrates (>60%) (20). Feng et al. (47) studied the HTC treatment of leftover steamed bread (LSB) and pitaya peel (PP), the surface area and pore volume values of LSB hydrochar were 3.5–17.2 times higher and 6.0–47.7 times higher than that of PP hydrochar, respectively. Due to the difference in main compositions, the starch in LSB was easier to be carbonized than the lignin and cellulose in PP. Pecchi et al (48) concluded that lipid-rich FW could produce a secondary char phase after HTC through ethanol extraction, which was the fuel precursor and could improve the coal-like properties of hydrochar. And carbohydrates and proteins-rich FW produced less secondary char while being rich in short-chain compounds.

### Physicochemical properties

The influencing factors of HTT are the key control points of this process, and the properties of products obtained from FW are significantly various under different HTT conditions. In terms of pH, with the rising HTC reaction severity, the pH increased, which was mainly attributed to the formation of low-pK_a_ organic structures and soluble organic alkalis, and the degradation of intermediated organic acid (15, 21–23). In terms of elemental composition, with the rising HTC reaction severity, the content of C increased by 10% and the content of N decreased (15). The decrease in N content was owing to the hydrolysis of amino acids and proteins through HTC (49). High temperatures (>170°C) are favorable for P accumulation on hydrochar (15). After the HTC of Chinese cabbage residue, the H/C and O/C ratios decreased by 11–25% and 46–63%, respectively, which indicated that hydrochar had increasing aromaticity and coal-like properties (50). With the increase of HTC reaction severity, the content of ${\text{NO}}_{3}^{--}$N increased, while the concentrations of ${\text{PO}}_{4}^{3--}$P and ${\text{NH}}_{4}^{+}$-N reduced (15). In terms of functional groups, the hydrochar surface could generate abundant function groups after the HTC of FW (15). Saha et al. (21) found that with the increase in HTC temperature, the hydrochar surface formed more acidic functional groups. The functional groups on the hydrochar surface were various with the changes in HTC conditions, which were mainly attributed to the degradation of hemicellulose, cellulose, and lignin (19). In terms of fuel properties, Yan et al. (51) carried out the HTC treatment of FW to produce solid fuel with an ignition temperature close to lignite. In the SCWG experiments, the H_2_-rich syngas production increased and the removal efficiencies of TOC and COD were enhanced with the increase in temperature. Studies showed that high volatile content could promote the reactivity of HTC reaction of FW, which could make hydrochar achieve greater fuel combustion and lower combustibility index (51–53). The HTT conditions have significant effects on the pH, elemental composition, functional groups, and fuel properties.

### Structural properties

The type of FW can usually influence the morphologies and structures of the products during the process of HTC. In addition, HTT conditions have significant effects on FW properties. HTT temperature has greater effects on the changes of FW properties than HTT retention time (15, 47). Saqib et al. (19) and Sharma et al. (24) found that with the increase of HTC strength (HTC temperature or duration) the surface morphology significantly changed. The effect of temperature was even more significant. This was that the degraded products of organic components were more easily dissolved into the liquid phase at high temperatures. With the increasing HTC reaction severity, the surface of hydrochar showed undulated structures, holes, and carbonaceous spheres successively, which were uniformly distributed in hydrochar. The FW primarily consists of sugars and carbohydrates. During the process of HTC, carbohydrates can form direct carbon microspheres, while sugars can also produce spherical carbonaceous material after the processes of degradation, polymerization, and condensation reactions (54).

## Application of hydrothermal treatment to food waste

The HTC, HTL, and SCWG are promising technologies to dispose of FW to produce energy and resource materials through changing their nutritional compositions, and physicochemical properties. The application of HTT on FW is summarized in Supplementary Table S2. It can be seen that HTC is more common for FW treatment. Feng et al. (47) studied the HTC of leftover steamed bread and pitaya peel, and this hydrochar was used as an adsorbent to remove rare earth ions from wastewater. Sharma et al. (24) prepared energy-intensive pelletization by HTC of FW and found that using molasses as a binder could obtain the best energy yield. Tradler et al. (20) converted restaurant food waste into hydrochar with high fuel qualities under the HTC conditions of 200°C for 6 h, which was used for co-combustion. Wang et al. (15) used Chinese cabbage residues to prepare hydrochar under the HTC conditions of 180, 200, 220, and 240°C for 2–6 h. And they found that the hydrochar had dissolved organic compounds and abundant nutrients but reduced phytotoxicity to be an optimal medium for plants' seedlings and growth. Yan et al. (51) combined HTC and SCWG to convert FW into both a hydrochar fuel and an H_2_-rich syngas. They found that the volatile matter of hydrochar was lower and the fixed carbon was higher than that of food waste. And the heating value of hydrochar was 22.68 MJ/kg under the HTC condition of 275°C for 60 min, which was close to lignite. When the condition of SCWG of HTC process water was 480°C for 45 min, the hydrogen conversion efficiency was 46.91%, the carbon conversion efficiency was 35.05%, and the removal efficiencies of TOC and COD were 83.04 and 82.99%, respectively. Su et al. (55) used HTC to treat FW for fuel application. The fuel ratio (FC/VM) was 0.112–0.146 and the higher heating value was 21.13–24.07 MJ/kg. He et al. (56) carried out a co-HTC process of FW and wet yard waste with an acid catalytic reaction for bioenergy application. They found that catalytic hydrochar had superior attributes in terms of comprehensive combustion behavior, including the HHV could reach 25 MJ/kg with citric acid catalysis and the highest utilization efficiency of carbon was 97.5%. The HTL of food waste can be applied to the production of bio-oil. Studies have shown that HTL has been effectively applied to animal food waste, such as offal, carcasses, and fish processing residues (57); Other studies report HTL on fruit and vegetable processing residues (58, 59). Stablein et al. (60) carried out the HTL of food waste to produce bio-oil and studied the effects of process parameters on bio-oil quality. Some studies have shown that SCWG was suitable for treating wet FW and related wastewater, for example, fruit waste, kitchen waste, food effluent, municipal waste leachate, sewage sludge, and so on (61–64).

## Economic analyses

The HTT of FW is feasible on a laboratory scale, however, pilot-scale or large-scale technologies are limited due to the high-pressure conditions of the process and the high cost of investment (65). The HTC has attracted more attention for commercialization compared to HTL and SCWG, which is probably due to the milder operating conditions. To carry out commercial technology products based on a laboratory scale, it is necessary to evaluate the technical feasibility and economic viability, for example, experimental process parameters, mass and energy balances, detailed reactor design, process modeling, cost estimation, and discounted cash flow analysis, and so on. Saqib et al. (66) listed the information about small enterprises with HTC technology on an industrial scale such as SunCoal, TerraNova Energy, and Ingelia S.L with a capacity of 8,000–50,000 tons of wet biomass per year. At present, some studies mainly focus on the economic evaluation of the HTT of biomass, while few studies focus on the economic evaluation of the HTT of FW. And those studies only define technical and economic assumptions based on their process designs and cost estimations, thus hindering comparisons between different production plants. Therefore, to make the economic assessments of different studies comparable, the US Department of Energy's BioEnergy Technologies Office (BETO) has presented an idea of the N^th^-Plant strategy to unify the key assumptions. To improve the commercial feasibility of HTT of FW, Marzbali et al. (65) put forward a few suggestions such as operating at a larger scale to achieve economies of scale, using a catalyst or blending to reduce the HTT process condition severity, and product functionalization.

## Challenges and future perspectives

According to the literature summarized in this mini-review, the HTT has significant effects on the properties of FW, which shows great advantages in the treatment of FW. Note that further studies to fill the current research gaps are needed to improve the application of HTT for FW treatment technically and economically, challenges, and perspectives coexist: (1) At present, the mechanism of HTT for FW has been investigated according to the changes of basic ingredients. However, there may be some interactions between intermediates or by-products, and these still require further study. (2) The application of HTT of FW has been proved to be feasible in lab-scale studies. However, few related industrial reactors have been built effectively. For pilot or practical applications, further detailed studies and evaluations should be carried out both technically and economically. (3) To better and large-scale application of HTT in the disposal and utilization of FW, the techno-economic analysis, overall energy balance, and life cycle assessment of the HTT process are necessary. In addition, the comparison and analysis of the above indexes between HTT and other treatment technologies (e.g., composting and pyrolysis) should be further studied.

## Conclusion

The annual production of FW is huge around the world. Among the traditional and emerging treatment methods, HTT is a more economical and energy-integrated process and can produce energy-dense fuels and valuable chemicals. This mini-review presents an overview of the latest development of HTT on FW. HTT includes HTC, HTL, and SCWG, and HTC is the most widely used in the treatment of FW. HTT can convert wet FW into more valuable products quickly and efficiently, which can avoid energy consumption during the drying process. The advantages, disadvantages, and mechanisms of HTT on FW have been summarized. The influence factors of HTT on FW include composition, HTT temperature, HTT residence time, and so on, among which composition and temperature are the main influential factors. HTT temperature has greater effects on the changes of physicochemical and structural properties than those of HTT retention time. The effects of HTT on the nutritional, physicochemical, and structural properties of FW have been widely focused on and studied. The nutritional composition of FW can be significantly impacted by HTT conditions so that the physical and chemical properties of FW can be changed. The type of FW can usually influence the morphologies and structures of the products during the process of HTC. The HTC can convert FW into hydrochar with high fuel quality and superior comprehensive combustion behavior. The HTL of FW can be applied to the production of bio-oil. The SCWG is suitable for treating the processing water of HTC of FW to generate syngas with a high H_2_. However, there are few studies on the economic analyses of FW by HTT. The challenges and future perspectives on HTT of FW have been summarized, that is, industrial reactors should be built effectively, and techno-economic analysis, overall energy balance, and life cycle assessment of the HTT process are necessary. In addition, by overcoming the bottleneck and difficulty in large-scale production, the preparation of high-value-added products from FW by HTT will be a promising commercial production.

## Author contributions

XZ: investigation, resources, writing original draft, reviewing and editing, conceptualization, software, and formal analysis. QQ: investigation and formal analysis. XS: conceptualization and formal analysis. WW: review, formal analysis, conceptualization, and supervision. All authors contributed to the article and approved the submitted version.

## Funding

This work was supported by the National Natural Science Foundation of China (Grant No. 51976110) and the Postdoctoral Innovation Project of Shandong Province (Grant No. 202101002).

## Conflict of interest

The authors declare that the research was conducted in the absence of any commercial or financial relationships that could be construed as a potential conflict of interest.

## Publisher's note

All claims expressed in this article are solely those of the authors and do not necessarily represent those of their affiliated organizations, or those of the publisher, the editors and the reviewers. Any product that may be evaluated in this article, or claim that may be made by its manufacturer, is not guaranteed or endorsed by the publisher.
