# Supplementary material for: Hydrothermal treatment: An efficient food waste disposal technology
Source: Front Nutr. 2022 Sep 12;9:986705. doi: 10.3389/fnut.2022.986705 (PMC9512071; doi:10.3389/fnut.2022.986705)
Supplement: Supplementary file 1 [file Data_Sheet_1.DOCX]

**Additional files**

**Hydrothermal Treatment: A Efficient Food Waste Disposal Technology**

Xinyan Zhang ^a, *^, Qingyu Qin ^b^, Xun Sun ^c, *^, Wenlong Wang ^a^

*^a^ National Engineering Laboratory for Reducing Emissions from Coal Combustion, Engineering Research Center of Environmental Thermal Technology of Ministry of Education, Shandong Key Laboratory of Energy Carbon Reduction and Resource Utilization, School of Energy and Power Engineering, Shandong University, Jinan, Shandong, 250061, China*

*^b^ Laboratory of Biomass and Bioprocessing Engineering, College of Engineering, China Agricultural University, Beijing 100083, China*

*^c^* *Key Laboratory of High Efficiency and Clean Mechanical Manufacture, Ministry of Education, School of Mechanical Engineering, Shandong University, Jinan 250061, China*

Corresponding authors: **Xinyan Zhang**, Email: sddxzxy2020@sdu.edu.cn and **Xun Sun**, Email: [xunsun@sdu.edu.cn](mailto:xunsun@sdu.edu.cn)

**Table S1** Hydrothermal Treatment of Food Waste.

| **Treatment method** | **HTC** | **HTL** | **SCWG** |
| --- | --- | --- | --- |
| Advantages [1] | No limit on the water content of feedstock, produce high-quality solid char named hydrochar, producing nutrient-rich process water, sterilization effect, high process efficiency and dewatering capacity | Producing bio-oil or bio-crude, energy efficient | Producing hydrogen or methane, less heat loss |
| Disadvantages [1] | Liquid products are of complex composition, expensive and  complex reactors | High temperature, high pressure, expensive and complex reactors, high capacities water handling equipment | High temperature, high pressure, expensive and  complex reactors, high capacities water handling  equipment |
| Mechanisms | Six main procedures, including hydrolysis, dehydration, decarboxylation, condensation, polymerization, and aromatization | The process mechanism involves the hydrolysis of biopolymers into water-soluble oligomers followed by the breakup of intramolecular and intermolecular hydrogen bonds into simple monomers [2] | SCWG mainly occurs the following reactions, including hydrolysis, dehydration, hydration, dehydrogenation [3] |
| Processing treatments | HTC converts biomass into a valueadded product (solid fuel) at a comparatively low temperature (180-  250°C) and saturated pressure (2-10 MPa) [4, 5] | HTL requires an operating temperature of 250-370°C at 5-20 MPa for 5-60 min, wherein water is in the liquid phase [6] | SCWG uses water at a supercritical state in the range of 600-700^o^C to  generate mainly H_2_ and CO_2_ with/without a catalyst [7]. |

**Table S2** Application of Hydrothermal Treatment in Food Waste.

| **Treatment method** | **Material** | **Application** | **Reference** |
| --- | --- | --- | --- |
| HTC | Restaurant food waste | Fuel | [8] |
| HTC | Leftover steamed bread and pitaya peel | Wastewater adsorbent | [9] |
| HTC | Food waste | Energy-intensive pelletization | [10] |
| HTC | Food waste | Fuel | [11] |
| HTC | Chinese cabbage residues | Fertilizer | [12] |
| Co-HTC | Food waste and wet yard waste | Fuel | [13] |
| HTC and SCWG | Food waste | Hydrochar fuel, H_2_-rich syngas | [14] |
| HTL | Fruit and vegetable processing residues | Bio-oil | [15,16] |
| HTL | Animal food waste (offal, carcasses, fish processing residues) | Bio-oil | [17] |
| HTL | Food waste | Bio-oil | [18] |
| SCWG | Wet food waste and the related wastewater | H_2_-rich syngas, pollutants management | [19, 20,21, 22] |

**Reference**

[1] Kumar M, Oyedun AO, Kumar A. A review on the current status of various hydrothermal technologies on biomass feedstock. Renew Sustain Energ Rev (2018) 81:1742-1770. doi.org/10.1016/j.rser.2017.05.270

[2] Peterson AA, Vogel F, Lachance RP, Froling M, Antal JMJ, Tester JW. Thermochemical biofuel production in hydrothermal media: a review of sub- and supercritical water technologies. Energy Environ Sci (2008) 1:32-65. doi.org/10.1039/B810100K

[3] Madenoğlu TG, Sağlam M, Yüksel M, Ballice L. Hydrothermal gasification of biomass model compounds (cellulose and lignin alkali) and model mixtures. J Supercrit Fluids (2016) 115:79-85. doi.org/10.1016/j.supflu.2016.04.017

[4] Mumme J, Eckervogt L, Pielert J, Diakité M, Rupp F, Kern J. Hydrothermal carbonization of anaerobically digested maize silage. Bioresour Technol (2011) 102:9255-9260. doi.org/10.1016/j.biortech.2011.06.099

[5] Jain A, Balasubramanian R, Srinivasan MP. Hydrothermal conversion of biomass waste to activated carbon with high porosity: a review. Chem Eng J (2016) 283:789-805. doi.org/10.1016/j.cej.2015.08.014

[6] Barreiro DL, Prins W, Ronsse F, Brilman W. Hydrothermal liquefaction (HTL) of microalgae for biofuel production: state of the art review and future prospects. Biomass Bioenergy (2013) 53:113-127. doi.org/10.1016/j.biombioe.2012.12.029

[7] Yoshida Y, Dowaki K, Matsumura Y, Matsuhashi R, Li D, Ishitani H, et al. Comprehensive comparison of efficiency and CO2 emissions between biomass energy conversion technologies-position of supercritical water gasification in biomass technologies. Biomass Bioenergy (2003) 25:257-272. doi.org/10.1016/S0961-9534(03)00016-3

[8] Tradler SB, Mayr S, Himmelsbach M, Priewasser R, Baumgartner W, Stadler AT. Hydrothermal carbonization as an all-inclusive process for food-waste conversion. Bioresource Technology Reports (2018) 2: 77-83. doi.org/10.1016/j.biteb.2018.04.009

[9] Feng YF, Sun HJ, Han LF, Xue LH, Chen YD, Yang LZ, et al. Fabrication of hydrochar based on food waste (FWHTC) and its application in aqueous solution rare earth ions adsorptive removal: Process, mechanisms and disposal methodology. J Clean Prod (2019) 212:1423-1433. doi.org/10.1016/j.jclepro.2018.12.094

[10] Sharma HB, Panigrahi S, Dubey BK. Food waste hydrothermal carbonization: Study on the effects of reaction severities, pelletization and framework development using approaches of the circular economy. Bioresour Technol (2021) 333:125187. [doi.org/10.1016/j.biortech.2021.125187](https://doi.org/10.1016/j.biortech.2021.125187)

[11] Su H, Zhou X, Zheng R, Zhou Z, Zhang Y, Zhu G, et al. Hydrothermal carbonization of food waste after oil extraction pre-treatment: study on hydrochar fuel characteristics, combustion behavior, and removal behavior of sodium and potassium. Sci Total Environ (2021) 754:142192. doi.org/10.1016/j.scitotenv.2020.142192

[12] Wang MQ, Zhang MY, Chen XH, Chen A, Xiao R, Chen XP. Hydrothermal conversion of Chinese cabbage residue for sustainable agriculture: Influence of process parameters on hydrochar and hydrolysate. Sci Total Environ (2022) 812:152478. doi.org/10.1016/j.scitotenv.2021.152478

[13] He MJ, Zhu XF, Dutta S, Khanal SK, Lee KT, Masek O, Tsang DCW. Catalytic co-hydrothermal carbonization of food waste digestate and yard waste for energy application and nutrient recovery. Bioresource Technol. (2022) 344:126395. doi.org/10.1016/j.biortech.2021.126395

[14] Yan M, Liu JY, Yoshikawa K, Jiang JH, Zhang Y, Zhu GJ, et al. Cascading disposal for food waste by integration of hydrothermal carbonization and supercritical water gasification. Renew Energ (2022) 186: 914-926. doi.org/10.1016/j.renene.2022.01.049

[15] Chan YH, Yusup S, Quitain AT, Uemura Y, Sasaki M. Bio-oil production from oil palm biomass via subcritical and supercritical hydrothermal liquefaction. J Supercrit Fluids (2014) 95:407-412. doi.org/10.1016/j.supflu.2014.10.014

[16] Tekin K. Hydrothermal conversion of Russian olive seeds into crude bio-oil using a CaO catalyst derived from waste mussel shells. Energ Fuel (2015) 29:4382-4392. doi.org/10.1021/acs.energyfuels.5b00724

[17] Zheng JL, Zhu MQ, Wu HT. Alkaline hydrothermal liquefaction of swine carcasses to bio-oil. Waste Manag (2015) 43:230-238. doi.org/10.1016/j.wasman.2015.05.010

[18] Stablein MJ, Aierzhati A, Watson J, Si B, Zhang YH. Characterization and bioremediation potential of byproducts from hydrothermal liquefaction of food wastes. Bioresour Technol Report (2020) 12:100555. doi.org/10.1016/j.biteb.2020.100555

[19] Adar E, Ince M, Bilgili MS. Supercritical water gasification of sewage sludge by continuous flow tubular reactor: a pilot scale study. Chem Eng J (2020) 391:123499. doi.org/10.1016/j.cej.2019.123499

[20] Chen Y, He Y, Jin H, Guo L. Resource utilization of landfill leachate gasification in supercritical water, Chem Eng J (2020) 386:124017. doi.org/10.1016/j.cej.2020.124017

[21] Chen Y, Yi L, Li S, Yin J, Jin H. Catalytic gasification of sewage sludge in near and supercritical water with different catalysts. Chem Eng J (2020) 388:124292. doi.org/10.1016/j.cej.2020.124292

[22] Yan M, Su H, Zhou Z, Hantoko D, Liu J, Wang J, et al. Gasification of effluent from food waste treatment process in sub- and su- percritical water: H2-rich syngas production and pollutants management, Sci Total Environ (2020) 730:138517. doi.org/10.1016/j.scitotenv.2020.138517
